# Pregnancy Care during the COVID-19 Pandemic in Germany: A Public Health Lens

**DOI:** 10.3390/ijerph20032721

**Published:** 2023-02-03

**Authors:** Antonia Leiße, Julia Dötzer, Alice Ruhnau, Leona Aschentrup, Florian Fischer, Kamil J. Wrona

**Affiliations:** 1 School of Public Health, Bielefeld University, 33615 Bielefeld, Germany; 2 Faculty of Engineering and Mathematics, University of Applied Sciences Bielefeld, 33619 Bielefeld, Germany; 3 Faculty of Health, University of Applied Sciences Bielefeld, 33619 Bielefeld, Germany; 4 Institute of Public Health, Charité—Universitätsmedizin Berlin, 10117 Berlin, Germany

**Keywords:** maternal care, pregnancy, crisis, Corona, SARS-CoV-2

## Abstract

The COVID-19 pandemic has led to various challenges in German health care, including pregnancy care. This paper aims to provide an overview of the pandemic-related challenges faced by pregnant women, new mothers, and their families in maternal and newborn care. A literature review was performed by including international literature as well as recommendations of institutions and official stakeholders. These challenges refer to restrictions at all stages of pregnancy, including wearing masks during labour, limitations of a companion of choice during birth, and restrictions of unvaccinated women from attending, e.g., antenatal classes. Compared with the general population, COVID-19 vaccination of pregnant women was recommended later, as pregnant women were initially excluded from clinical trials. Women who gave birth during the COVID-19 pandemic also reported mental health issues. The findings stress the importance of the inclusion of pregnant women in clinical trials. This might also help to overcome vaccine hesitancy among pregnant women and women seeking family planning. Taking the COVID-19 pandemic as an example, one must weigh the changes and restrictions associated with the potential disadvantages for mothers, newborns, and their families in pregnancy care against the measures to control the pandemic.

## 1. Introduction

Three years after the first case of the coronavirus disease (COVID-19) was detected, different measures to control the pandemic are still part of people’s everyday lives. Because of the pandemic, people experienced restrictions that impacted their lives more or less. While wearing a face mask has been considered a new “normal”, other measures such as the closure of facilities have impacted individuals in a more severe way. All these political actions have altered the everyday lives of people, including pregnant women [1]. Far-reaching measures were also adopted in the health sector to protect it from overburden. The first shutdown in Germany led to a reduction of more than 40% in the number of elective surgeries [2]. Because maternal and newborn care, especially childbirth, cannot be postponed, pregnant women and their partners faced restrictions related to the antenatal and postpartum period [3]. Generally, pregnancy and having a baby are considered major events in women’s lives, as they experience various changes, including physical, psychological, and social changes [1,4]. During this time, women are particularly vulnerable to emotional instability and stress [5]. Therefore, expectant mothers are considered a group with special needs. Consequently, it is important to take this into account when planning appropriate measures, such as ensuring medical care. The COVID-19 pandemic has placed policymakers in a situation where decisions have to be made under uncertainty due to limited evidence and experience as well as under time pressure. For that reason, it is important to take a closer look at pandemic-related changes and the particularities that pregnant women are facing to gain knowledge and consider it for the future. From today’s perspective, previous measures, such as separating women confirmed to be SARS-CoV-2 positive from their newborns, are no longer performed unless there is a medical indication.

This commentary summarizes the available literature on pandemic-related challenges in pregnancy care with two main aims. Firstly, we want to provide a critical overview of the changes that occurred in pregnancy care as a result of the COVID-19 pandemic. Secondly, implications should be derived to improve pregnancy care—not only during crises but also in general. Thus, different actors can benefit from the experience gained in the last three years of the pandemic and adapt it to future similar scenarios. To do so, we adopt a public health lens, which enables consideration of the weighing of the benefits and harms under uncertainty and autonomy vs. commitment to act (on individual, societal, and political levels) in terms of prevention, as well as aspects of equity and equality. This commentary is based on a narrative synthesis of the international literature concerning the health of pregnant women during the COVID-19 pandemic. This evidence has been applied to the situation in Germany and serves as a starting point for further discussions.

## 2. Weighing the Benefits and Harms under Uncertainty

Since the outbreak of the COVID-19 pandemic in Germany, many different measures have been taken by the German government to contain the pandemic. These ranged from contact restrictions to industry closures to mandatory masking. All these measures were introduced without any explicit benefit being ensured [6]. Similarly, the introduction of vaccination against SARS-CoV-2 was highly debated. Nevertheless, vaccination was recommended by all authorities for the majority of the population [7]. In December 2020, BioNTech’s Comirnaty, the first vaccine against COVID-19, was licensed in Germany [8].

As there were not enough vaccines available for the entire population at the beginning of the outbreak, priority was given to vaccinating groups of people at high risk for severe or fatal courses, including people aged 80 years and older and people at increased risk of infection due to their occupation. As of June 2021, the entire population had the opportunity to be vaccinated [9]. Today (January 2023), around two years after the first vaccine in Germany [10], the vaccination rate of those who have received at least a single dose of a COVID-19 vaccine is 76.4% [11]. However, vaccination for pregnant women was a point of contention. As pregnant women were largely excluded from pivotal trials during vaccine development, there was little evidence of the safety and efficacy of the vaccine for this group [12]. For this reason, no specific vaccination recommendation was made for pregnant women until September 2021—about nine months after the start of the general vaccination campaign and two months after the end of prioritization [13]. Vaccination of pregnant women was recommended as there was growing evidence suggesting that vaccination protects both the expectant mother and the unborn child from the increased risk of severe disease progression and associated pregnancy complications [14,15]. Like the Standing Committee on Vaccination (STIKO), the German Society of Gynecology and Obstetrics (Deutsche Gesellschaft für Gynäkologie und Geburtshilfe e.V.) advised pregnant women to get vaccinated against SARS-CoV-2 with an mRNA vaccine beginning in the second trimester of pregnancy [13,16].

In the past, pregnant women were excluded from vaccine development for a long time. The rationale was that pregnant women bear the responsibility for another life [17]. Based on past experience, women are considered particularly susceptible to selected diseases, including COVID-19 [18]. Although, this is an ethically controversial decision; some authors call for the inclusion of pregnant women into clinical trials for the opportunity to receive a vaccine and protect themselves and their offspring [17,18]. For this reason, studies have now been conducted to test the effectiveness of COVID-19 vaccines for pregnant women and have proven that vaccination with an mRNA vaccine is effective and safe for pregnant women. These studies have also disproven the initially suspected increased numbers of stillbirths due to COVID-19 vaccines. In fact, a systematic review and meta-analysis conducted by Prasad et al. (2022) found a significant decrease in stillbirths and no adverse pregnancy outcomes (e.g., miscarriages) that could be attributed to COVID-19 vaccines in pregnancy. However, when interpreting the results, one must consider the heterogenous study situation, as four out of seven studies investigating the effect of COVID-19 vaccination on stillbirths showed lower odds of stillbirths in vaccinated individuals, whereas three studies found no or a negative effect on stillbirths in vaccinated individuals [19].

## 3. Autonomy vs. Commitment to Act

In addition to the public debate about the appropriateness of vaccination for pregnant women, the late recommendation of vaccination—not to mention the widespread misinformation—may have contributed to the uncertainty of pregnant women at an already stressful time. It would be particularly problematic if pregnant women were not vaccinated despite the available evidence demonstrating a positive benefit of vaccination [19,20]. Historically, vaccination is known as an important public health measure to fight infectious diseases [21]. At the beginning of the pandemic, the hope for containing the pandemic revolved around developing effective vaccines. Thus, the aim of these vaccines was to prevent COVID-19 indirectly (by vaccinating people in contact with at-risk patients) or directly (by vaccinating people themselves) [22]. Now, considering the high incidences caused by e.g., the omicron variant, it is apparent that re-infections cannot be prevented [23]. However, vaccinations retain the potential to prevent severe disease progression at the individual level. Consequently, the individual-level potential impacts the population level, as health systems are less burdened as a result of reducing severe disease progression and thus preventing hospitalizations [24,25]. Though vaccines are considered important on the individual and population levels, people have the right to refuse vaccination. To increase vaccination coverage, different measures have been implemented. These refer to restrictions, sanctions, and bans for those individuals who do not meet one of the 3G [26]. In Germany, for example, the so-called “3G” rule was implemented, which excluded individuals from different public venues who did not meet the requirement of being vaccinated (*geimpft*) or recovered (*genesen*) from COVID-19 or showing a current negative test (*getestet*) [27]. The question arises regarding to what extent these restrictions limit autonomy, as vaccinated people enjoy greater freedoms compared to their unvaccinated counterparts who do not meet the requirement of being currently negatively tested or recovered from COVID-19. According to the Corona Protection Ordinance of 9 December 2021, vaccination or booster vaccination status was often a prerequisite for participation in public life during pandemic periods. This also applies to participation in antenatal classes with a face-to-face format. Depending on the design of the contact restrictions, pregnant women without vaccination or booster status are sometimes excluded from corresponding offers [28]. Given that pregnant women were excluded in the development process of the COVID-19 vaccine at first, women and especially pregnant women are more often vaccine-hesitant. Studies indicate that the prevalence of unvaccinated pregnant women and their vaccine hesitancy is quite high [29,30,31]. For example, the study of Ercan et al. (2022) investigated COVID-19 vaccine hesitancy in pregnant women in Turkey prior to the availability of a vaccine. The results suggested that 73.2% of the study sample would not accept a COVID-19 vaccination even though it was considered safe and effective in pregnancy [32]. The reasons for vaccine hesitancy are, e.g., concerns about side effects on the unborn child and on the pregnant women themselves, whereas a higher educational level and an older age are associated with a greater COVID-19 vaccine acceptance [32,33,34]. However, especially in the context of pregnancy and COVID-19, vaccine hesitancy might be evoked by misinformation regarding the impact of vaccines on fertility and reproductive health [35]. This experience highlights the importance of clear, easy-to-understand communication of health information. With the flood of different, sometimes dubious sources, it is even more important to be particularly present as an official, government-commissioned provider of information, such as the Robert Koch Institute in Germany (RKI). At the political level, the different specifications and implementation of measures in different federal states have contributed to the uncertainty. Accordingly, it would be beneficial for the federal states to adopt uniform measures. At the lowest level, institutions, companies, and other actors have been given a lot of freedom to implement measures, which makes collective knowledge of existing restrictions in society almost impossible.

As pandemic-related measures apply not only to the general population but also to pregnant women and women giving birth, pandemic-related measures had an impact on women from pregnancy to the postpartum period. During the prenatal period, 26.1% of women reported difficulties in attending routine antenatal visits during the pandemic in Germany [36]. According to the available literature, most restrictions were experienced during childbirth and postpartum. For example, a study conducted in Japan indicated that women were requested to wear a mask during labour and delivery [37]. Moreover, a study on maternal and newborn care in selected European countries, including Germany, revealed limitations in birth experiences due to the pandemic. These limitations refer to restrictions on a companion of choice being present during birth and restrictions on mobility in labour [36]. However, according to recommendations by the World Health Organization (WHO) on pregnancy and childbirth during COVID-19, a positive childbirth experience includes having a companion of choice during birth and the possibility to change position during labour [38]. The positive effects of a companion being present during birth refer to the practical, emotional, and informational support that can be provided by a companion [39]. Additionally, the Corona Protection Ordinance in Germany stipulates that mothers and their newborn children may only receive one person per day for one hour. The family must, therefore, choose and may only be visited by one particular person [40]. These restrictions impact not only new mothers but also their partners. Andrews et al. (2022) examined the impact of the restrictions on maternal care from a father’s point of view. The results highlight that fathers lack important memories of their child that are considered important not only for developing feelings of fatherhood but also for bonding with the newborn [23]. This needs to be stressed, as fatherhood shifted from men being financial providers and being considered as having a less active part in child nurturing in the past, and a group of so-called new fathers spent more time with their children [41]. Moreover, women who are suspected or confirmed to be COVID-19-positive experienced further restrictions. These refer to contact limitations to the newborn, e.g., reducing skin-to-skin contact and breastfeeding [42]. According to life course models, the health of an individual is particularly shaped by experiences had in life, including those had in childhood [43]. As mothers that gave birth during the COVID-19 pandemic are more likely to experience breastfeeding problems because of higher odds of acute stress levels [44], their children are unable to profit from its advantages. These potential disadvantages should be emphasized, because breastfeeding is associated with various (health) benefits, including reduced risk of infectious and chronic diseases during the life course as well as better development of children’s cognitive function [45,46]. From the current point of view, the WHO does not recommend these contact limitations for COVID-19-positive women as the benefits of breastfeeding and skin-to-skin contact outweigh the risk of transmission [38].

## 4. Equity and Equality

There are populations that are more exposed to the virus and some that are at significantly greater risk of long-term (mental) health damage. This leads to discussions about vulnerability, which includes pregnant women, a group that has been less in the focus of these discussions so far. To promote equity, it is essential to target these particularly vulnerable groups, considering not only their physical vulnerability but also their mental health.

In March 2022, the WHO published a scientific brief on the impact of the pandemic on mental health. With the inclusion of the Global Burden of Disease (GBD) and an umbrella review, the WHO concluded that the pandemic and the accompanying public health and social measures have increased mental health problems worldwide [47]. On the basis of the GBD findings, it was emphasized that the pandemic reinforced the need to improve mental health systems in most countries [48]. A rapid review by Mauz et al. (2021) investigating the mental health of the German population during the COVID-19 pandemic reports inconclusive results. Although the authors report an overall very resilient adult population with stable mental health, they point out the need to study particularly vulnerable groups [49]. Because of their specific life situation and the changes described above, one could assume that pregnant women could belong to one of these vulnerable groups. Several international studies have dealt with the topic and concluded that there are increasing mental problems among expectant mothers [5,50,51,52]. A study by Preis et al. (2020) suggests that the current pandemic is exacerbating stress-related vulnerabilities that predict heightened anxiety [53]. The authors further differentiate two forms of stress in pregnant women during the COVID-19 pandemic: the feeling of being unprepared for birth due to the pandemic is described as “Preparedness Stress”, while “Perinatal Infection Stress” refers to the anxiety of being infected with the virus. Many other publications refer to the Canadian population, for which an increase in depression and anxiety has been reported in particular [12,54,55]. A cross-national review also reports a significant increase in both symptoms. In addition, COVID-19-specific concerns, especially about the health of the unborn child in pregnant women, are reported in Italy and Turkey [50]. The previously described circumstances during birth in times of COVID-19 were also examined more closely concerning their impact on mental health. For instance, a study conducted by Mayopoulos et al. (2021) examined the birth experiences of primarily American women who gave birth before and during the COVID-19 pandemic. The results indicated that women who gave birth during the pandemic have greater odds of experiencing acute stress levels in comparison to women that gave birth before the pandemic. This higher acute stress response to childbirth was associated with childbirth-related post-traumatic stress disorder (PTSD), bonding, and breastfeeding problems [44]. Childbirth-related PTSD is associated with different consequences, such as negative emotions of the mother towards the child and parenting stress. Risk factors for childbirth-related PTSD are, e.g., a negative birth experience, lack of social support, and pre-existing maternal psychiatric problems [56,57,58].

Thus, international studies provide evidence that some mental disorders increased during the pandemic and that COVID-19- and pregnancy-specific concerns were compounded. However, studies are currently lacking that assess the extent to which the mental health of pregnant women in Germany changed during the pandemic. Moreover, this essay stresses the importance of mental health during pregnancy, with a special focus on pandemic periods. Being pregnant and giving birth under pandemic conditions can be seen as a stressor. Generally, and not taking the pandemic into account, pregnancy is considered an emotional period in women’s lives. Especially in the postpartum period, women are at a high risk of having depression [59]. As mentioned above, a companion being present during labour and birth can provide practical, emotional, and informational support [39]. Since social contact is seen as a protective factor to reduce the risk of depression [60], restrictions on social contact should be strongly weighed and only implemented with reference to existing evidence.

Generally, there is still a lack of studies, especially in the area of mental health, that explicitly refer to the target group of pregnant women in Germany. The public health measures we have experienced and are currently experiencing affect all of us, albeit to different degrees for different groups. Mental health is ultimately influenced by social determinants, e.g., family situation, culture, and socioeconomic status [61]. These determinants must, therefore, be given greater importance in future studies. Finally, relationship problems have also increased during the COVID-19 pandemic, which in turn could affect a woman’s mental health during pregnancy [55]. In order to gain sufficient insight into the mental health of pregnant women during the pandemic in Germany, it is important to consider aspects that influence one’s mental health sufficiently. Promoting equity means addressing the neglected and most vulnerable populations. For this reason, there is a need for greater action to shed light on the specific needs of pregnant women during the COVID-19 pandemic.

## 5. Conclusions

This essay gives insights into the peculiarities, measures, and resulting changes that pregnant women were facing during the COVID-19 pandemic. From the current point of view and according to the recommendations of the WHO (2021) on positive childbirth experiences, women who gave birth during the pandemic experienced great limitations, especially during birth, as there were restrictions on a companion being present during birth and they were requested to wear a mask [36,37]. However, one must note that COVID-19 is considered the most serious pandemic in modern times [62] and the measures were, therefore, mandatory, as little was known about the virus. Accordingly, some lessons can be learned from the current situation, as some measures should be taken to be prepared for future pandemics. Nevertheless, all measures must be weighed against the risk of infection with the described consequences for mothers and their families, which may be different in each pandemic situation.

When analysing the impact of the COVID-19 pandemic on mothers, one must note that the possibilities for comparing exposed and unexposed women are limited, as the pandemic affects all pregnant women equally in Germany. Therefore, further research is needed that focuses on comparing women who experienced restrictions during pregnancy and birth due to the pandemic and women who did not.

Based on this critical literature review, our research calls for a better consideration of pregnant women. This includes the integration of pregnant women in clinical trials, as COVID-19-related health risks are also prominent in pregnant women and vaccines are considered effective in them as well. Furthermore, one must consider that the exclusion of pregnant women in trials may also influence the acceptance or hesitancy of COVID-19 vaccination. To increase the vaccination rate of pregnant women, it is mandatory for relevant stakeholders to address misconceptions and be better networked with different stakeholders. A reasonable cross-level information system, for example, is a possible approach. This means that there should be consistent communication from the political level to doctors, nurses, and midwives.

A companion being present during birth can offer advantages for mothers, their newborns, and the whole family. One approach to prevent the transmission of SARS-CoV-2 on the one hand and to offer women support on the other hand could be to expand internet services in hospitals and make them available free of charge to enable all women to have digital contact with their acquaintances. Even if this cannot be a substitute, it could be a possible alternative during the temporary period of a pandemic. With the discontinuation of offers in times of pandemic, including birth classes, women should still be given the opportunity to prepare for childbirth and parenthood. This can take place in the form of online offers and courses [63].

## Data Availability

Not applicable.
